# Expression of Bone Morphogenetic Protein-2 in the Chondrogenic and Ossifying Sites of Calcific Tendinopathy and Traumatic Tendon Injury Rat Models

**DOI:** 10.1186/1749-799X-4-27

**Published:** 2009-07-21

**Authors:** Pauline Po Yee Lui, Lai Shan Chan, Yau Chuk Cheuk, Yuk Wa Lee, Kai Ming Chan

**Affiliations:** 1 Department of Orthopaedics and Traumatology, Faculty of Medicine, The Chinese University of Hong Kong, Hong Kong SAR, PR China; 2 The Hong Kong Jockey Club Sports Medicine and Health Sciences Centre, Faculty of Medicine, The Chinese University of Hong Kong, Hong Kong SAR, PR China

## Abstract

**Background:**

Ectopic chondrogenesis and ossification were observed in a degenerative collagenase-induced calcific tendinopathy model and to a lesser extent, in a patellar tendon traumatic injury model. We hypothesized that expression of bone morphogenetic protein-2 (BMP-2) contributed to ectopic chondrogenesis and ossification. This study aimed to study the spatial and temporal expression of BMP-2 in our animal models.

**Methods:**

Seventy-two rats were used, with 36 rats each subjected to central one-third patellar tendon window injury (C1/3 group) and collagenase-induced tendon injury (CI group), respectively. The contralateral limb served as controls. At week 2, 4 and 12, 12 rats in each group were sacrificed for immunohistochemistry and RT-PCR of BMP-2.

**Results:**

For CI group, weak signal was observed at the tendon matrix at week 2. At week 4, matrix around chondrocyte-like cells was also stained in some samples. In one sample, calcification was observed and the BMP-2 signal was observed both in the calcific matrix and the embedded chondrocyte-like cells. At week 12, the staining was observed mainly in the calcific matrix. Similar result was observed in C1/3 group though the immunopositive staining of BMP-2 was generally weaker. There was significant increase in BMP-2 mRNA compared to that in the contralateral side at week 2 and the level became insignificantly different at week 12 in CI group. No significant increase in BMP-2 mRNA was observed in C1/3 group at all time points.

**Conclusion:**

Ectopic expression of BMP-2 might induce tissue transformation into ectopic bone/cartilage and promoted structural degeneration in calcific tendinopathy.

## Background

Calcific tendinopathy is a poorly characterized tendon degenerative disorder that is extremely common in athletes as well as in the general population with repetitive tendon overuse. Despite its prevalence, its underlying pathogenesis is poorly understood and treatment is usually symptomatic. Recently, we reported the presence of chondrocyte phenotype and ectopic ossification in a collagenase-induced patellar tendon injury model [1]. Erroneous differentiation of healing tendon fibroblasts might account for failed healing and ossification in the model [1]. A lower chance and extent of ectopic chondrogenesis and ossification were observed after traumatic patellar tendon traumatic injury which healed with reduced cellularity, vascularity and reorganization of extracellular matrix. (Lui PPY, Cheuk YC, Fu SC, Chan KM: Chondrometaplasia and Ossification During Repair of Patella Tendon Injury, submitted) This suggested similar biological pathway might be activated in both traumatic and collagenase-induced tendon injuries. The extent of injury might determine the healed or fail-healing status, consistent with failed healing in tendinopathy was due to the accumulation of micro-injuries that the tendon failed to resolve.

Bone morphogenetic proteins are multi-functional growth factors that belong to the TGF-beta superfamily [2]. They have strong effect on bone and cartilage growth as well as with important roles during embryonic pattern and early skeletal formation. To date, around 20 BMP family members have been identified. BMP-2 is among the most studied member of the family and has been used in many studies for augmentation of bone and bone-tendon junction regeneration [3,4]. Because of the role of BMP-2 in bone formation, it is commonly found in bone and is generally absent in tendon. We hypothesized that ectopic expression of BMP-2 contributed to ectopic chondrogenesis and ossification in our animal models. This study aimed to report the spatial and temporal expression of BMP-2 protein and mRNA in both animal models.

## Methods

This study was approved by the Animal Research Ethics Committee of the authors' institution.

### Traumatic tendon injury model

Thirty-six Sprague-Dawley male adult rats (6–8 weeks, average body weight of 300 g) were used [5]. Under general anesthesia, an incision was made to expose the patellar tendon. The central one-third of the patellar tendon (1 × 4 mm) from the distal apex of the patellar to the insertion of the tibial tuberosity was then removed and the gap was left open. The wound was then closed in layers. Sham operation was performed in the contralateral limb and served as control.

### Collagenase-induced injury

Thirty-six male Sprague Dawley rats, (8 weeks, weight 200–250 grams) were used [1]. After anesthesia with 2.5% pentobarbital (4.5 mg/kg body weight), hairs over the lower limb were shaved. Patellar tendon was located by positioning the knee at 90°. Twenty microliters (0.015 mg/μl in 0.9% saline, i.e. 0.3 mg) of bacterial collagenase I (Sigma-Aldrich, St Louis, MO) or saline were injected into the patellar tendon intratendinously with a 30G needle in one limb while the contralateral limb was injected with saline [6].

### Sample harvest

The rats with different surgical procedures were allowed free cage movement immediately after surgery. At week 2, 4 and 12 after injury, the rats were sacrificed and the patellar tendons in both limbs were harvested (n = 12). Six samples were used for immunohistochemical staining of BMP-2 and the other six samples were used for real time RT-PCR.

### General histology and immunohistochemistry

The patellar tendon was washed in PBS, fixed in buffered formalin and 100% ethanol, embedded in paraffin, cut longitudinally to 5-μm thick sections and mounted on 3-aminopropyl-triethoxy-silane (Sigma-Aldrich, St Louis, MO) coated slides. After deparaffination, the sections were stained with haematoxylin and eosin. Immunohistochemistry was done as described previously [1,5]. Briefly, after removal of paraffin, the sections were rehydrated, decalcified, quenched of endogenous peroxidase activity and subjected to antigen retrieval. After blocking with 5% normal donkey serum, the sections were stained with specific antibodies against BMP-2 (Santa Cruz Biotechnology, Santa Cruz, CA; 1:100) at 4°C overnight. Donkey anti-goat horseradish peroxidase (HRP)-conjugated secondary antibody (Santa Cruz Biotechnology; 1:100) was then added for an hour, followed by 3,3' diaminobenzidine tetrahydrochloride (DAKO, Glostrup, Denmark) in the presence of H_2_O_2_. Afterwards, the sections were rinsed, counterstained in hematoxylin, dehydrated with graded ethanol and xylene, and mounted with p-xylene-bis-pyridinium bromide (DPX) permount (Sigma Aldrich, St Louis, MO). Primary antibody was replaced with blocking solution in the controls. For good reproducibility and comparability, all incubation times and conditions were strictly controlled. The sections were examined under light microscopy (Leica DMRXA2, Leica Microsystems Wetzlar GmbH, Germany).

### Quantitative real-time RT-PCR

The patellar tendon was harvested and homogenized for RNA extraction with Trizol reagent (Gibco BRL, Life Technologies, Invitrogen, Carlsbad, CA). The RNA was reverse transcribed to cDNA by the First Strand cDNA kit (Promega, Madison, WI). The primer sequences and annealing temperature for BMP-2 and -actin were shown in Table 1. The real-time PCR machine, the reaction kits, and the software used in the experiments were purchased from Roche (LightCycler, Roche Diagnostics GmbH, Penzbergh, Germany). The expression of BMP-2 was normalized to the expression of β-actin gene. Relative gene expression of the operated limb to the control limb was calculated according to the 2^-ΔΔCT ^formula.

**Table 1:** Table showing the primer sequences and annealing temperature of target genes

Gene	Primer sequences	Annealing temperature
BMP-2	Forward: 5'-TAGTGACTTTTGGCCACGACG-3'	58°C
	Reverse: 5'-GCTTCCGCTGTTTGTGTTTG-3'	
β-actin	Forward: 5'-ATCGTGGGCCGCCCTAGGCA-3'	52°C
	Reverse: 5' TGGCCTTAGGGTTCAGAGGGG-3'	

### Data analysis

The immunohistochemical data was qualitatively described. The mRNA data was presented in box-plots. To compare the mRNA level among different time points, Kruskal-Wallis test followed by post-hoc comparison of different time points with control using Mann-Whitney U test was performed. To compare the mRNA level of injury groups with the time-matched controls, Wilcoxon signed-rank test was used. All the data analysis was done using SPSS (SPSS Inc, Chicago, IL, version 16.0). p < 0.05 was regarded as statistically significant.

## Results

### Immunohistochemistry of BMP-2

**Figure 1:**
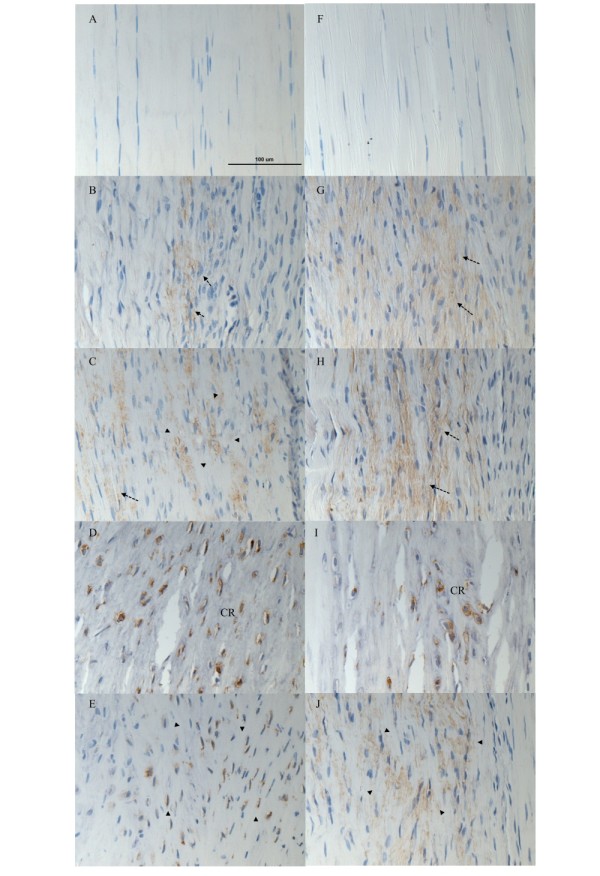
**Immunohistochemical staining of BMP-2 in collagenase-induced calcific tendinopathy and central one-third traumatic injury model**. (A-E) collagenase-induced calcific tendinopathy and (F-I) central one-third patellar tendon traumatic injury models at different time points. A: week 12 saline-injection control; B: week 2; C: week 4; D-E: week 12 after collagenase injection. F: week 12 sham control; G: week 2; H: week 4; I-J: week 12 after central one-third traumatized injury. For the collagenase-induced calcific tendinopathy group, weak signal was observed at the tendon matrix at week 2. At week 4, matrix around chondrocyte-like cells was also stained. At week 12, the staining was observed mainly in the calcific matrix and the matrix around chondrocyte-like cells. Similar result was observed in the central one-third patellar tendon traumatic injury group though the immunopositive staining of BMP-2 was generally weaker. At weeks 2 and 4, weak signal was observed in the tendon cell matrix. At week 12, the matrix around chondrocyte-like cells and the calcific matrix were stained. No immunopositivity of BMP-2 was observed in both control groups. arrows: tendon cells; arrowheads: chondrocyte-like cells; CR: calcific region; Magnification: 400×; error bar: 100 μm

No immunopositivity of BMP-2 was observed in both control groups (Figure 1A and 1F). For the collagenase-induced calcific tendinopathy model, weak signal was observed at the tendon matrix at week 2 (Figure 1B, arrows). At week 4, tendon matrix was still stained (Figure 1C, arrows) and matrix around chondrocyte-like cells was also stained (Figure 1C, arrowheads), consistent with the time of appearance of chondrocyte-like cells in this animal model. In one sample, calcification was observed and BMP-2 signal was observed both in the chondrocyte-like cells embedded in calcific matrix and the surrounding matrix. At week 12, the staining was observed mainly in chondrocyte-like cells within the calcific matrix in all samples (Figure 1D, CR) and chondrocyte-like cells in uncalcific matrix (Figure 1E, arrowheads).

Similar result was observed in the central one-third traumatic injury model though the immunopositive staining of BMP-2 was generally weaker. At weeks 2 and 4, weak signal was observed in the tendon cell matrix in 6/6 samples (Figure 1B, arrows) and 5/6 samples, (Figure 1B, arrows) respectively. The signal at week 4 was stronger than that at week 2. At week 12, the matrix around the chondrocyte-like cells was stained in 3/3 samples (Figure 1J, arrowheads). The calcific matrix and the embedded chondrocyte-like cells (Figure 1I, arrowheads) were stained in samples with calcific deposits. The overall immunopositive staining of BMP-2 decreased at week 12.

### mRNA expression of BMP-2

**Figure 2:**
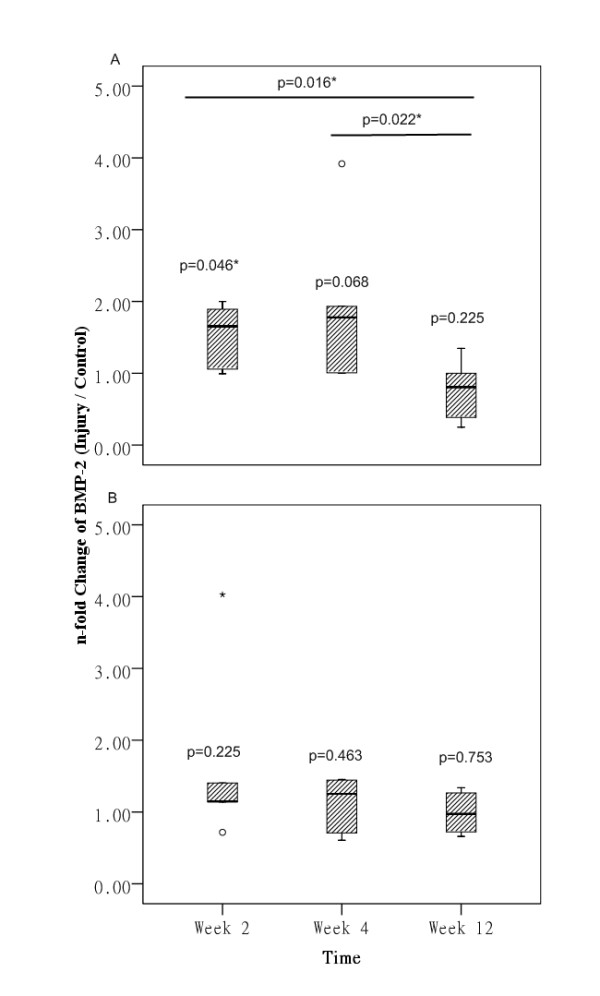
**mRNA expression of BMP-2 in collagenase-induced calcific tendinopathy and central one-third traumatic injury model**. (A) collagenase-induced calcific tendinopathy; (B) central one-third patellar tendon traumatic injury models at week 2, 4 and 12. * indicated p < 0.05.

For the collagenase-induced calcific tendinopathy group, there was significant increase in mRNA expression of BMP-2 compared to that at the contralateral side at week 2 (p = 0.046) (Figure 2A). There was also increase in mRNA expression of BMP-2 at week 4 but it was marginally insignificant (p = 0.068). The mRNA level became insignificantly different from that at the contralateral side at week 12 (p = 0.225). There was significant difference in mRNA level of BMP-2 between week 2 and week 4 with week 12 (overall: p = 0.021; post-hoc comparison: week 2 vs week 12: p = 0.016; week 4 vs week 12: p = 0.022).

For the central one-third traumatic injury group, there was no significant difference in BMP-2 mRNA expression compared to that at the contralateral side despite the increase at week 2 and week 4 (all p > 0.05) (Figure 2B).

## Discussion

The pathogenesis of calcific tendinopathy, including the cause of tendon pain, tendon degeneration and calcification, remained largely unknown and hence current treatments are usually symptomatic. Change of tendon loading due to mechanical overload, compression or disuse have been implicated as the possible etiologies [7], but they do not completely explain the cellular and molecular alternations seen in the diseased tendon such as chondrometaplasia and ectopic ossification, hypercellularity, vascularity and extracellular matrix degeneration. Ectotopic chondrogenesis and ossification have been reported in our established patellar calcific tendinopathy rat model and to a lesser extent, in the traumatic patellar tendon injury model [1]. (Lui Cheuk YC, Fu SC, Chan KM: Chondrometaplasia and Ossification During Repair of Patella Tendon Injury, submitted) We hypothesized that ectopic expression of BMP-2 might be involved in the chondrometaplasia and ossification in both models. This study aimed to study the spatial and temporal expression of BMP-2 protein and mRNA in both animal models.

BMP-2 protein was detected in the chondrocyte-like cells and calcific deposits in both injury models but not in control samples, indicating that BMP-2 might be involved in the pathogenesis of ectopic chondrogenesis and ossification. There was also increase in BMP-2 mRNA at week 2 and week 4 in both injury models though it was statistically significant only at week 2 for the collagenase-induced calcific tendinopathy group. This was consistent with previous clinical study which reported ectopic overexpression of BMPs in the subacromial bursa and it was suggested to account for the chondrogenic transformation and ectopic mineralization of rotator cuff tendon in patients [8]. The expression of BMP-2 in the chondrocyte-like cells and calcific deposits suggested that BMP-2 might be involved in ectopic chondrogenesis and ossification. This was supported by the reported role of BMP-2 in promoting chondrocyte differentiation, osteoblast differentiation and endochondral ossification [9,10]. The insignificant difference in mRNA expression in both models might be due to the large sample variation, particularly for the traumatic injury group which showed only 50% calcification rate and at a much lower extent, and the expression became more focal, localizing mainly at the chondrocyte-like cells and calcific deposits, at week 12 in both models. As the mRNA expression was calculated based on total cells, this might dilute the expression at week 12. Care therefore should be taken when interpreting the mRNA data and studying the expression also at the protein level by immunohistochemistry is suggested in tissue samples.

As we observed earlier expression of BMP-2 mRNA and protein at week 2 in healing tendon cells, before the time of its appearance in chondrocyte-like cells and calcific deposits, this also supported that calcific tendon degeneration is mediated by the healing tendon cells which have plasticity and are under erroneous cell differentiation due to the changes in the mechanical and biological microenvironment. Indeed, injection of rhBMP-2 into tendon increased ectopic bone formation, indicating that tendon consisted of cells that were responsive to BMP-2 and were capable of differentiating along the chondro-osseous pathway [11]. Another study also reported that BMP could induce transdifferentiation of tenocytes into chondrocytes in vitro [12]. Arthritic synovial membranes have also been shown to express BMP-2 & BMP-6 and could influence cell turnover [13].

We observed lower level of expression of BMP-2 at similar chondrogenic and ossification sites in the traumatic tendon injury model. This agreed with the lower degree and extent of ectopic chondrogenesis and ossification in the model and further supported the role of BMP-2 in ectopic chondrogenesis and ossification.

Regarding the possible changes in mechanical and biological microenvironment that cause the ectopic expression of BMP-2 in tendons, it is currently not clear. Small leucine-rich repeat proteoglycans such as biglycan and fibromodulin were reported to regulate the differentiation of tendon progenitor cells into chondrocytes and bone cells through modulating the BMP-2 signaling pathway [14]. In their study, tendon progenitor cells from biglycan- and fibromodulin- knockout mice formed bone in addition to tendon-like tissue after transplantation in vivo, whereas wild type tendon progenitor cells only formed tendon-like tissue [14]. There was increased sensitivity of tendon progenitor cells from biglycan- and fibromodulin- knockout mice to BMP-2 stimulation with increased phosphorylation of Smad1, Smad5 and Smad8 as well as more abundant nuclear localization of phosphorylated Smad1 than those of wild type cells [12]. Changes in the composition of the extracellular matrix might affect the cellular response of healing tendon cells and promote their differentiation to osteoblasts and chondroblasts rather than tenoblasts.

In addition to BMP-2, other members of the TGF-beta superfamily such as BMP-4, BMP-7 and TGF-beta 1, may also induce tissue transformation into ectopic bone/cartilage and promoted structural degeneration in calcific tendinopathy. Previous studies have shown that BMP-4 was involved in cutaneous [15] and muscle ossification [16]. BMP-4 and -7 and TGF-beta1 were also reported to be involved in the initiation and development of ossification of spinal ligaments (OSL) [17]. Activities of BMPs are inhibited extracellularly by BMP-binding proteins such as Noggin and Chordin as well as intracellularly by Smad6, tob and Smurf1 [2]. Information on the expression of these osteogenic factors and BMP antagonists, in addition to the expression of BMP-2, will give a more comprehensive picture of the osteogenic signals contributing to the regulation of ectopic chondrogenesis and ossification in calcific tendinopathy.

## Conclusion

In conclusion, we reported the expression of BMP-2 in tendon cells, chondrocyte-like cells and calcific deposits in the calcific tendinopathy animal model, and to a lesser extent, in the traumatic window injury model, which might account for the chondrometaplasia and ectopic ossification. Further studies are required to understand the causes for increased expression of BMP-2 and the role of BMP-2 signaling pathway in tendon cell differentiation and tendon degeneration.

## Competing interests

The authors declare that they have no competing interests.

## Authors' contributions

PPYL designed the study, performed statistical analysis and interpret the results and draft the manuscript. LSC, YCC, YWL carried out the animal operation, immunohistochemical staining and RT-PCR and analyzed the data. KMC designed the study and draft the manuscript. All authors read and approved the final manuscript.
